# The contribution of transdiagnostic vulnerability factors in patients with chronic insomnia

**DOI:** 10.3389/fpsyt.2023.1162729

**Published:** 2023-04-03

**Authors:** Mojtaba Habibi Asgarabad, Hoda Doos Ali Vand, Pardis Salehi Yegaei, Farzaneh Hooman, Reza Ahmadi, Chiara Baglioni, Shahram Moradi

**Affiliations:** ^1^Health Promotion Research Center, Iran University of Medical Science, Tehran, Iran; ^2^Department of Psychology, Norwegian University of Science and Technology, Trondheim, Norway; ^3^Department of Health Psychology, School of Behavioral Sciences and Mental Health (Tehran Institute of Psychiatry), Iran University of Medical Sciences, Tehran, Iran; ^4^Positive Youth Development Lab, Human Development & Family Sciences, Texas Tech University, Texas, TX, United States; ^5^Center of Excellence in Cognitive Neuropsychology, Institute for Cognitive and Brain Sciences, Shahid Beheshti University, Tehran, Iran; ^6^Department of Clinical Psychology, School of Medicine, Shahid Beheshti University of Medical Sciences, Tehran, Iran; ^7^Department of Psychology, Ahvaz Branch, Islamic Azad University, Ahvaz, Iran; ^8^Department of Clinical Psychology, Faculty of Medicine, Zanjan University of Medical Sciences, Zanjan, Iran; ^9^Human Sciences Department, University of Rome Guglielmo Marconi Rome, Rome, Italy; ^10^Department of Psychiatry and Psychotherapy, Medical Faculty, University of Freiburg, Freiburg, Germany; ^11^Department of Health, Social and Welfare Studies, Faculty of Health and Social Sciences, University of South-Eastern Norway, Porsgrunn, Norway

**Keywords:** insomnia, transdiagnostic factors, anxiety, depression, perfectionism, emotion regulation, repetitive thinking

## Abstract

**Introduction:**

Various transdiagnostic factors have been associated with insomnia severity. The current study aimed to predict insomnia severity based on a group of transdiagnostic factors including neuroticism, emotion regulation, perfectionism, psychological inflexibility, anxiety sensitivity, and repetitive negative thinking after controlling for depression/anxiety symptoms and demographic characteristics.

**Methods:**

Two hundred patients with chronic insomnia disorder were recruited from a sleep disorder clinic. Participants completed the Insomnia Severity Index (ISI), Clinical Perfectionism Questionnaire (CPQ), Acceptance and Action Questionnaire-II (AAQ-II), Anxiety Sensitivity Index-3 (ASI-3), Repetitive Thinking Questionnaire (RTQ-10), Big Five Inventory (BFI-10), Emotion Regulation Questionnaire (ERQ), and Depression Anxiety Stress Scale (DASS-21).

**Results:**

After controlling for the confounding variables (depression/anxiety symptoms and demographic characteristics), hierarchical multiple linear regression suggested the significant association of neuroticism (BFI), cognitive reappraisal (ERQ), personal standards (CPQ), evaluative concerns (CPQ), physical concerns (ASI), cognitive concerns (ASI), and repetitive negative thinking (RTQ) with insomnia severity.

**Discussion:**

The findings support the role of transdiagnostic factors, especially physical concerns, repetitive negative thinking, and neuroticism in chronic insomnia. Future research using longitudinal designs is required to verify the causal status of transdiagnostic variables.

## 1. Introduction

Insomnia refers to the dissatisfaction with sleep quality or quantity, leading to clinically significant distress or impairments in significant areas of functioning (1). Specifically, this disorder pertains to the difficulty maintaining sleep, characterized by frequent awakenings or problems returning to sleep after awakening (1). High rates of comorbidity between chronic insomnia and other medical and psychiatric disorders have been found (2, 3). Two disorders that commonly occur along with insomnia disorder are anxiety and depression. A prospective study on the general population found that insomnia was closely linked with depression and anxiety (4), the result that was replicated in a large body of evidence [e.g., (5–9)]. As such, the effect of sociodemographic characteristics on insomnia severity has been documented in various studies. For instance, lower education level was shown to be a risk factor for psychological and social stressors and as a result, leads to insomnia (10–12). Insomnia was also found to be more common in women and older adults [(13); e.g., (10)]. From these evidence, one may conclude that the role of psychological transdiagnostic factors on insomnia severity might be affected or confounded by the individual’s level of depression/anxiety symptoms and demographic characteristics.

In recent years, research and treatment has focused more on the transdiagnostic approach (14). Despite the “disorder-focused” approach that focuses exclusively on a single disorder to understand its etiology and develop its interventional protocol (15), transdiagnostic approach concentrates on factors that are common among comorbid disorders (16). The common transdiagnostic factors that their association with insomnia were well documented include emotion dysregulation (17), neuroticism (18), psychological inflexibility (19), perfectionism (20), anxiety sensitivity (21), and repetitive negative thinking (22).

Maladaptive emotion regulation strategies have been identified as underlying mechanisms in insomnia (23). In their overview of literature, Cerolini et al. (24) found that empirical research highlighted the vital role of emotion dysregulation in the insomnia onset and maintenance, the result that was replicated in another review study (25). Davoodi et al. (26) also investigated the predictor role of various transdiagnostic factors on insomnia, finding that greater difficulties in emotion regulation was a significant contributor to this sleep–wake disorder.

Another transdiagnostic factor implicated in insomnia is neuroticism, also known as negative affectivity (27),which refers to the tendency to experience negative affects, so that people with high neuroticism can hardly maintain their calmness when they are emotionally aroused (28). A study on general population indicated that a greater level of neuroticism in young adults is longitudinally related to the onset of insomnia (29). Gurtman et al. (30) showed that the intensity of insomnia increases with the increase in neuroticism.

Psychological inflexibility can also play a role in the onset and maintenance of insomnia (19). Psychological inflexibility is characterized as “the rigid dominance of psychological reactions over chosen values and contingencies in guiding action” (31). Kato (32) found that psychological inflexibility contributes to insomnia severity, beyond the effect of neuroticism, and could mediate the relation between neuroticism and insomnia severity.

Perfectionism is another transdiagnostic factor associated with insomnia disorder. Maladaptive or clinical perfectionism is characterized by the fear of making mistakes (personal standards), perceiving unrealistic expectations by others and the fear of negative evaluation and rejection by society associated with not being perfect (evaluative concerns) (33). Individuals with chronic insomnia are likely to show more maladaptive perfectionism than healthy controls (34). In addition, greater levels of perfectionism was found to have a positive link with insomnia severity in university students (35).

Another important transdiagnostic factor associated with insomnia is anxiety sensitivity, which refers to the trait-like fear of feelings and sensations related to anxiety (36). A higher level of anxiety sensitivity has been associated with longer sleep onset latency (i.e., longer time taken to fall asleep) (37). The physical concerns dimension of anxiety sensitivity, which refers to the belief that any bodily sensation due to emotional arousal can lead to physical diseases, was shown to have a moderating role in the relationship of sleep anticipatory anxiety with sleep onset latency (38). The cognitive concerns dimension of anxiety sensitivity, defined as the belief that physical symptoms of anxiety lead to serious psychological disorders, was associated with daytime impairments such as fatigue, in people with chronic insomnia (39).

Repetitive negative thinking is another important transdiagnostic factor, present across various disorders, including insomnia (22, 40, 41). Repetitive negative thinking refers to the process in which cognitive representation of one or multiple psychological stressors becomes chronically or repetitively activated (42). Rumination and worry are the two best-known aspects of repetitive thought (43), that have significant associations with polysomnographic indices of disrupted sleep (44), and sleep disturbances, both in healthy people (45), and in insomnia patients (46).

There is clear evidence to support the relationships between the transdiagnostic factors and chronic insomnia (18, 19, 21); however, the extent to which each of these factors can affect insomnia remains unclear. Assessing such transdiagnostic factors together is helpful to estimate the contribution of each of these factors, among others, on chronic insomnia. Hence, the current study aimed to examine the prediction of insomnia severity based on the six candidate transdiagnostic factors including (1) clinical perfectionism, (2) psychological inflexibility, (3) anxiety sensitivity, (4) repetitive negative thinking, (5) neuroticism, and (6) emotion dysregulation, after controlling for the confounding effects of demographic characteristics and depression/anxiety symptoms. Based on established research findings, we hypothesized that the abovementioned transdiagnostic factors would be positively associated with insomnia severity. However, no directional hypothesis was given on the magnitude of the contribution of each factor, since no previous study had included the present variables at the same time in a predictive model of insomnia severity, making it difficult to say which one has a larger weight in association with insomnia.

## 2. Materials and method

### 2.1. Participants

Two hundred and seventeen patients with chronic insomnia disorder were recruited from sleep disorders clinic of Rasoul Akram Hospital in Tehran. Inclusion criteria included: (a) a minimum age of 18 years, (b) fluency in Persian language, and (c) meeting the diagnostic criteria for chronic insomnia disorder (such as difficulty initiating or maintaining sleep, early-morning awakening with inability to fall asleep again, and the presence of the sleep disturbance for at least 3 months) based on the Diagnostic and Statistical Manual, Fifth Edition [DSM-5; (1)]. The exclusion criteria were having: (a) severe mental disorders (e.g., bipolar disorder and psychosis), (b) brain injury, (c) substance abuse, (d) other sleep disorders (e.g., sleep apnea and restless legs syndrome), and (e) a rotating shift work within the last year Structured Clinical Interview for DSM-5 Axis I Disorders (SCID-5) were used to determine the presence of insomnia. In the case of a suspicion of an occult sleep disorder, individuals underwent polysomnography.

### 2.2. Measures

Due to the large number of included variables in the present regression model, the following battery of well-stablished and widely used brief measures were selected since they have shown that could maintain the validity and reliability of their long versions.

#### 2.2.1. The structured clinical interview for DSM-5 axis I disorders (SCID-5)

The SCID-5 (47) is a standardized diagnostic interview for classifying mental disorders according to DSM-5 criteria. The Persian version of the SCID-5 had adequate test–retest reliability, ranging from 0.60 to 0.79, and the Kappa reliability was between 0.57 and 0.72 (48).

#### 2.2.2. Insomnia severity index

This is a seven-item self-report index developed by Bastien et al. (49) to assess insomnia severity and its three empirically derived subscales of impact, intensity, and satisfaction during the past two weeks. Items are scored on a 0–4 scale, with the total Insomnia severity index (ISI) ranging from 0 to 28. A score of 0–7 demonstrates “no clinically significant insomnia”; 8–14 shows “subthreshold insomnia”; 15–21 demonstrates “moderate clinical insomnia”; and 22–28 shows “severe clinical insomnia.” The *Cronbach’s* alpha of the Persian version of the ISI in patients with insomnia has been reported as 0.82 (50), and was 0.71 in this study.

#### 2.2.3. Depression anxiety stress scale (DASS-21)

This 21-item scale (51) is a short version of the DASS-42 (52); it was developed to measure the severity of depression, anxiety, and stress symptoms during the previous week, with 7 items for each subscale. Answers are scored on a four-point *Likert* scale, ranging from 0 (*did not apply to me at all*) to 3 (*applied to me very much or most of the time*). In the Iranian population, the test–retest coefficients have been reported as 0.85 for stress, 0.77 for depression, and 0.89 for anxiety, with respective internal consistency values of 0.90, 0.91, and 0.84 (53). In this study, the *Cronbach’s* alpha for depression, anxiety, and stress subscales were 0.80, 0.75, and 0.77, respectively.

#### 2.2.4. Emotion regulation questionnaire

The emotion regulation questionnaire (ERQ) was designed by Gross and John (54) to evaluate two emotion regulation strategies, namely expressive suppression (four items) and cognitive reappraisal (six items). Answers are based on a seven-point *Likert* scale ranging from 1 (*strongly disagree*) to 7 (*strongly agree*). In the Iranian population, the internal consistency for cognitive reappraisal and suppression has been reported as 0.76 and 0.72, respectively (55). In the present study, *Cronbach*’s alphas were 0.77 for expressive suppression and 0.84 for cognitive reappraisal.

#### 2.2.5. Big five inventory

This 10-item inventory was developed by Rammstedt and John (56) to measure 5 major personality traits: extroversion, agreeableness, conscientiousness, neuroticism, and openness, with 2 items for each subscale. It is measured on a seven-point *Likert* scale ranging from 1 (*strongly disagree*) to 7 (*strongly agree*). The Persian version of the Big five inventory (BFI-10) has appropriate test–retest reliability (*r* = 0.84) and acceptable internal consistency (57). The *Cronbach*’s alpha of 0.45 was obtained for BFI-10 in this study.

#### 2.2.6. Acceptance and action questionnaire-II

This seven-item questionnaire (31) was designed to assess psychological inflexibility or rigid reactions, such as avoiding or suppressing undesirable thoughts and feelings, or evaluating feelings negatively. Answers are based on a seven-point Likert scale ranging from 1 (*never true*) to 7 (*always true*), where higher scores indicate less psychological flexibility. Split-half coefficients and *Cronbach*’s alpha values of the Persian version of the Acceptance and action questionnaire-II (AAQ-II) ranged between 0.71 and 0.89 in different samples (58). *Cronbach*’s alpha for the AAQ-II was 0.63 in this study.

#### 2.2.7. Clinical perfectionism questionnaire

Clinical perfectionism questionnaire (CPQ) (33) is a 12-item questionnaire developed to measure clinical perfectionism, by determining the extent to which the people’s lives are affected by perfectionism. This questionnaire has two subscales: personal standards and evaluative concerns. Scoring is based on four-point *Likert* scales, ranging from 1 (*not at all*) to 4 (*all the time*). In an Iranian sample, the *Omega* reliability of CPQ was between 0.73 and 0.86 for men and women (59). We found satisfactory internal consistency for the CPQ total score (*α* = 0.57), personal standards (*α* = 0.62), and evaluative concerns (*α* = 0.60).

#### 2.2.8. Anxiety sensitivity index-3

This 18-item Index (60) measures three dimensions of anxiety, namely physical (*α* = 0.84), cognitive (*α* = 0.77), and social concerns (*α* = 0.84), with 6 items per subscale. Participants are asked to rate their concerns regarding the potential negative consequences of anxiety-related experiences, using five-point *Likert* scales ranging from 0 (*very little*) to 4 (*very much*). The internal consistency of the Anxiety sensitivity Index-3 (ASI-3) in the Iranian population was previously reported as 0.90 (61). In this study, these values were 0.84, 0.77, and 0.84 for physical, cognitive, and social concerns, respectively.

#### 2.2.9. Repetitive thinking questionnaire

The repetitive thinking questionnaire (RTQ-10) (62) is a unidimensional scale for measuring repetitive thinking. The RTQ-10 is rated on a 5-point scale, where 1 represents “*not at all true*,” 3 indicates “*somewhat true*,” and 5 reflects “*very true*.” The Persian version of the RTQ-10 was reported as having high internal consistency [α = 0.77; (63)]. The RTQ-10 also had adequate internal consistency (*α* = 0.89) in the present study.

### 2.3. Procedure

The sample consisted of individuals who were seeking treatment at the sleep disorders clinic of Rasoul Akram hospitals in Tehran. Individuals who volunteered for the study were first asked to fill written informed consent forms. They were informed that their participation in the study was completely voluntary. In addition, they were assured about the confidentiality and that their data would only be used for research purposes. Then, they underwent a structured clinical interview by a sleep clinician to verify inclusion criteria. Patients with the probability of an occult sleep disorder, underwent PSG to screen the physiological parameters related to a wide range of sleep disturbances (exclusion criterion “d”). Those who were not excluded during this phase, were asked to complete the following questionnaires: ISI, DASS-21, ERQ, BFI-10, CPQ, AAQ-II, ASI, and RTQ-10. Responding to the battery of questionnaires took 20–30 min and the order of questionnaires was varied randomly among respondents (questionnaire rotation) in order to increase the response accuracy. This study was approved by the ethics committee at the Iran University of Medical Sciences (approval code = IR.IUMS.REC.1397.1346).

### 2.4. Statistical strategies

The data screening included consistency checks and descriptive and graphical analysis checks. We decided whether to remove or keep outliers using the comparison between the mean and the 5% trimmed mean (*p* > 0.05). The substantive main study findings were not affected by the outliers’ presence or absence. Hence, the data of all participants were preserved and the original data were analyzed (64, 65). For data entry and statistical analyses, SPSS version 28 software was used. The Pearson correlation coefficient was used to indicate the relationship between variables. Hierarchical multiple linear regression was subsequently employed to indicate the predictive power of the included variables. Correlations were interpreted as having small (0.10), medium (0.30), large (0.50), or very large (0.70) effect sizes (66).

## 3. Results

The majority of participants were 18–45-year-old females (90.8%, *Mean _age_* = 29.67, *SD* = 12.41). Regarding marital status, 62.2% were single, 33.6% were married, and 4.2% were divorced/widowed. Regarding education level, 10.1% had completed elementary school, 12.9 had a diploma, 60.8% had a bachelor’s degree, and 16.1% had a master’s and/or PhD degree.

### 3.1. Correlation of insomnia severity with demographic and transdiagnostic factors

As shown in Table 1, ISI total score had a significant positive correlation with age (*r* = 0.14, *p* < 0.05), depression (DASS-21) (*r* = 0.58, *p* < 0.01), anxiety (DASS-21) (*r* = 0.76, *p* < 0.01), cognitive reappraisal (ERQ) (*r* = 0.73, *p* < 0.01), expressive suppression (ERQ) (*r* = 0.75, *p* < 0.01), neuroticism (BFI) (*r* = 0.70, *p* < 0.01), AAQ total score (*r* = 0.80, *p* < 0.01), CPQ total score (*r* = 0.13, *p* < 0.05), personal standards (CPQ) (*r* = 0.86, *p* < 0.01), evaluative concerns (CPQ) (*r* = 0.86, *p* < 0.01), physical concerns (ASI) (*r* = 0.47, *p* < 0.01), cognitive concerns (ASI) (*r* = 0.61, *p* < 0.01), social concerns (ASI) (*r* = 0.63, *p* < 0.01), and RTQ total score (*r* = 0.80, *p* < 0.01). In addition, total ISI score had a significant negative correlation with education level (*r* = −0.21, *p* < 0.01).

**Table 1:** Descriptive statistics and correlations of insomnia severity with demographics characteristics, and transdiagnostic vulnerability factors in insomnia.

Variables	*N*/Mean (SD)	Skewness	Kurtosis	Range (min-max)	Insomnia severity	Impact-ISI	Severity-ISI	Satisfaction-ISI
Age (years)	29.67 (12.41)				0.14^*^	−0.04	0.27^**^	0.09
Gender (0 = Female, 1 = Male)	197, 20				−0.04	−0.10	0.02	−0.04
Marital status (1 = Married, 2 = Single, 3 = Divorced/ Widow)	135, 73, 9				−0.05	−0.01	−0.07	−0.03
Education level (0 = Elementary School, 1 = Diploma, 2 = Bachelor, 3 = Master and PhD)	22,28,132,35				−0.21^**^	0.03	0.35^**^	0.15^*^
Job status (0 = Unemployed, 1 = Employed)	112, 105				−0.04	−0.14^*^	−0.20^**^	−0.04
Neuroticism (BFI-10)	5.90 (2.22)	0.10	−1.16	8 (2–10)	0.70^**^	0.55^**^	0.57^**^	0.64^**^
Physical concerns (ASI-3)	14.75 (5.93)	−0.7	−0.11	24 (0–24)	0.47^**^	0.45^**^	0.27^**^	0.42^**^
Cognitive concerns (ASI-3)	11.59 (5.73)	0.20	−0.58	24 (0–24)	0.61^**^	0.52^**^	0.49^**^	0.46^**^
Social concerns (ASI-3)	12.30 (6.23)	0.14	−0.91	24 (0–24)	0.63^**^	0.49^**^	0.55^**^	0.51^**^
CPQ	26.92 (4.85)	−0.36	−0.34	52 (12–37)	0.13^*^	0.26^**^	−0.08	0.20^**^
Personal standards (CPQ)	18.36 (6.29)	−0.62	−0.72	26 (1–27)	0.86^**^	0.73^**^	0.64^**^	0.75^**^
Evaluative concerns (CPQ)	11.77 (2.96)	−0.92	0.46	14 (2–16)	0.86^**^	0.74^**^	0.63^**^	0.76^**^
Cognitive reappraisal (ERQ)	24.77 (9.01)	−0.10	−0.54	47 (6–53)	0.73^**^	0.59^**^	0.57^**^	0.67^**^
Expressive suppression (ERQ)	18.11 (5.58)	−0.47	−0.10	24 (4–28)	0.75^**^	0.62^**^	0.58^**^	0.65^**^
Depression (DASS-21)	12.20 (4.72)	−0.28	−0.55	21 (0–21)	0.58^**^	0.50^**^	0.42^**^	0.48^**^
Anxiety (DASS-21)	14.74 (3.62)	−1.23	1.58	20 (1–21)	0.76^**^	0.67^**^	0.54^**^	0.67^**^
AAQ-II	38.59 (13.88)	−0.32	−0.70	52 (8–60)	0.80^**^	0.63^**^	0.63^**^	0.70^**^
RTQ	32.77 (8.76)	−0.52	−0.89	33 (12–45)	0.80^**^	0.65^*^	0.64^**^	0.72^**^

^*^p < 0.05, ^**^p < 0.01; N, number of participants; SD, standard deviation; ISI, insomnia severity scale; BFI, big five inventory; ASI-3, anxiety sensitivity index-3; CPQ, clinical perfectionism, questionnaire; ERQ, emotion regulation questionnaire; DASS, depression anxiety stress scale; AAQ-II, acceptance and action questionnaire-II; RTQ, repetitive thinking questionnaire.

### 3.2. Prediction of insomnia severity based on demographic and transdiagnostic factors

A hierarchical linear multiple regression model was performed to test the contribution of demographic and transdiagnostic factors in the prediction of insomnia severity. Demographic characteristics such as gender, age, education level, job status, and marital status were considered as the first block, with depression and anxiety as the second block. The third block comprised of emotion regulation strategies, neuroticism, psychological inflexibility, clinical perfectionism subscales, anxiety sensitivity dimensions, and repetitive negative thinking.

The first hierarchical regression analysis model (*R^2^* = 0.08), performed to assess the predictability of insomnia severity based on demographic characteristics, showed that only education level (*β* = 0.20, *p* < 0.01) was significantly able to predict insomnia severity. Gender (*β* = −0.06, *p* > 0.05), age (*β* = 0.04, *p* > 0.05), job status (*β* = −0.13, *p* > 0.05), and marital status (*β* = 0.01, *p* > 0.05) did not significantly predict insomnia severity. After controlling for demographic in the second model (*R^2^* = 0.10, *R^2^*Δ = 0.007), depression (*β* = 0.10, *p* > 0.05) and anxiety (*β* = 0.03, *p* > 0.05) were not significant predictors of insomnia severity. In the third model, depression was revealed as a significant predictor of insomnia severity (*β* = 0.34, *p* < 0.001). Finally, as seen in Table 2, after controlling for demographic characteristics, depression and anxiety in the third model (*R^2^* = 0.40, *R^2^*Δ = 0.30), the following transdiagnostic variables significantly predicted insomnia severity: cognitive reappraisal (ERQ) (*β* = 0.19, *p* < 0.01), neuroticism (BFI) (*β* = 0.24, *p* < 0.001), evaluative concerns (CPQ) (*β* = 0.16, *p* < 0.05), personal standards (CPQ) (*β* = 0.22, *p* < 0.01), physical concerns (ASI) (*β* = 0.34, *p* < 0.001), cognitive concerns (ASI) (*β* = 0.18, *p* < 0.05), and repetitive negative thinking (*β* = 0.33, *p* < 0.001). However, expressive suppression (ERQ) (*β* = 0.13, *p* > 0.05), psychological inflexibility (*β* = 0.07, *p* > 0.05), and social concerns (ASI) (*β* = −0.02, *p* > 0.05) did not significantly predict insomnia severity.

**Table 2:** Hierarchical multiple linear regression analyses for insomnia severity in insomniac sample.

Predictor variables	Regression 1β (se)	Regression2 β (se)	Regression3 β (se)	Tolerance	VIF
Gender	−0.06 (1.12)	−0.08 (1.13)	−0.04 (0.97)	0.89	1.12
Education level	0.20^**^ (0.43)	0.19^*^ (0.44)	0.25^**^ (0.44)	0.74	1.33
Age	0.04 (0.03)	0.05 (0.38)	0.17^*^ (0.03)	0.65	1.52
Job status	−0.13 (0.62)	0.12 (0.62)-	−0.12 (0.58)	0.96	1.03
Marital status	0.01 (0.55)	0.01 (0.55)	0.05 (0.49)	0.90	1.10
Depression (DASS-21)		0.10 (0.08)	0.34^***^ (0.08)	0.57	1.73
Anxiety (DASS-21)		0.03 (0.09)	−0.10 (0.09)	0.56	1.78
Neuroticism (BFI-10)			0.24^***^ (0.18)	0.55	1.80
Physical concerns (ASI)			0.34^***^ (0.06)	0.44	2.27
Cognitive concerns (ASI)			0.18^*^ (0.07)	0.42	2.37
Social concerns (ASI)			−0.02 (0.06)	0.51	1.92
Personal standards (CPQ)			0.22^**^ (0.07)	0.64	1.56
Evaluative concerns (CPQ)			0.16^*^ (0.12)	0.63	1.56
Cognitive reappraisal (ERQ)			0.19^**^ (0.04)	0.57	1.73
Expressive suppression (ERQ)			0.13 (0.06)	0.56	1.77
AAQ-II			0.07 (0.04)	0.34	2.88
RTQ			0.33^***^ (0.05)	0.29	3.44
*R*	0.28	0.32	0.63		
*R* ^2^	0.08	0.10	0.40		
adj *R*^2^	0.05	0.06	0.34		
*R* ^2^_Δ_	0.08	0.007	0.30		
*F*_Δ_	3.15^**^	0.75	9.94^***^		
*F*_model_	3.15^**^	2.64^**^	7.02^***^		

^*^p < 0.05, ^**^p < 0.01, ^***^p < 0.001; β, standardized regression coefficient; se, standard error; VIF, variance inflation factor; DASS, Depression Anxiety Stress Scale; BFI, Big Five Inventory; ASI-3, Anxiety Sensitivity Index-3; CPQ, Clinical Perfectionism Questionnaire; ERQ, Emotion Regulation Questionnaire; AAQ-II, the Acceptance and Action Questionnaire-II; RTQ, Repetitive Thinking Questionnaire. Dependent Variable = Insomnia severity, In Hierarchical regression procedure, the blocks were applied as = Block 1 = Predictors = Gender, education level, age, job status, and marital status; Block 2 = Predictors = Depression, anxiety, after adjusted for gender, education level, age, job status, marital status; Block 3 = Predictors = Neuroticism, physical concerns, cognitive concerns, social concerns, personal standards, evaluative concerns, expressive suppression, AAQ, RTQ, after adjusted for gender, education level, age, job status, marital status, depression, anxiety.

## 4. Discussion

The present study sought to identify the best predictive transdiagnostic factors of chronic insomnia in a sample of patients with chronic insomnia. The results demonstrated that all of the transdiagnostic variables evaluated in the current study had moderate to high correlations with insomnia severity, with the exception of clinical perfectionism (*r* = 0.13). Demographic characteristics, depression, and anxiety symptoms accounted for 10% of the variance of the ISI. Furthermore, 30% of insomnia severity variance was explained by the transdiagnostic factors (in order of highest to lowest coefficients) of physical concerns (ASI), repetitive negative thinking (RTQ), neuroticism (BFI), personal standards (CPQ), cognitive reappraisal (ERQ), cognitive concerns (ASI), and evaluative concerns (CPQ). In addition, physical concerns (ASI) and repetitive negative thinking (RTQ) served simultaneously as the most significant contributing variables for insomnia severity.

Both cognitive reappraisal (ERQ) and expressive suppression (ERQ) indicated significant correlations with insomnia severity, which is consistent with previous findings (67, 68). However, only cognitive reappraisal (ERQ) played a significant role in insomnia severity, which emphasizes the exclusive effect of emotion regulation strategies on insomnia severity. One possible explanation might be that although cognitive reappraisal during the day can regulate emotions *via* problem solving and negative affect management, it may play a negative role at bedtime by increasing sleep onset latency and reducing sleep quality (69).

Neuroticism was shown to be another factor that had a key role in the etiology of insomnia severity; which is in line with previous findings (see (70, 71)). High levels of neuroticism reflects negative emotions, guilt, moodiness, and poor active control in a person, leading to pre-sleep cognitive distortions, such as worrying, and active thinking at bedtime, which can be associated with over-excitation and hypersensitivity (30). This lack of capability to stop or manage thoughts at night can lead to insomnia (72).

Psychological inflexibility indicated a high significant correlation with insomnia severity (*r* = 0.80), which is consistent with previous studies (73, 74). Active “experiential avoidance” from experiencing symptoms or cognitions related to sleep problems may lead to increase in pre-sleep arousal and delay in falling asleep (75). Nevertheless, our study indicates that in the presence of other transdiagnostic factors which may be better risk factors of insomnia severity, psychological inflexibility could not be significantly predict insomnia.

This study, in line with previous studies (76, 77), found a significant but weak association between perfectionism and insomnia severity. Also, its two components of personal standards and evaluative concerns significantly contributed to insomnia severity. Maladaptive perfectionism may lead to heightened insomnia through its association with excessive arousal (78) and chronic fatigue (79), which are thought to be amplifying factors for insomnia (80, 81). Furthermore, perfectionists with chronic insomnia unrealistically expect to have excellent quality sleep and become extremely anxious or frustrated with any sleep deprivation. This may cause them to experience negative emotions, leaving them emotionally aroused, which can interfere with their sleep (82).

The present findings suggest that two anxiety sensitivity dimensions – namely physical and cognitive concerns – could predict insomnia severity, with physical concerns (ASI) carrying the highest weight among all the present transdiagnostic variables. This association is consistent with previous studies (83, 84). According to Harvey’s (72) cognitive model, people with elevated anxiety sensitivity are more alert to the physical and cognitive signs and symptoms associated with poor sleep and are more likely to discover these signs and symptoms. As a result, they catastrophize these cues as predictors of insufficient sleep, an inability to fall asleep, or low levels of daily performance, which in turn, heighten a person’s arousal and increase the likelihood of sleep disturbance. In contrast to physical and cognitive concerns, social concerns failed to contribute to insomnia severity. In general, the previous findings on the sub-dimension of social concerns are scarce, perhaps because the body of research has concentrated only on physical and cognitive concerns [e.g., (38)].

The study findings indicated that repetitive negative thinking had a strong correlation with insomnia severity. It was also a significant risk factor of insomnia severity, which is consistent with previous studies (44, 85). According to Harvey’s (72) cognitive model of insomnia, excessive worry and rumination (two components of repetitive negative thinking) about sleep, as well as long-term unresolved problems experienced during the day and night, result in attentional bias to sleep-related negative information, which trigger psychological arousal, and subsequently delay falling asleep.

### 4.1. Study limitations and future directions for research

Although our study provides new insights into the transdiagnostic factors that play crucial roles in insomnia severity, several limitations should be noted when drawing conclusions from our data. First, self-report measures are exposed to bias, owing to common method variance (CMV; (73)). Other methods, including actigraphy or daily sleep diaries, are suggested as alternatives, as well as conducting longitudinal studies to limit the effect of potential CMV biases [see (86)]. Second, examining a clinical sample limits the generalizability to other samples; thus, the findings must be interpreted cautiously. It is also preferable to compare the results between an insomniac and a non-clinical sample. Third, the majority of study participants were women. Although insomnia is more common in women than in men (13), we suggest that future studies consider equal number of men and women in their sample. This may increase the generalizability of their results to both male and female patients with insomnia and also provide the opportunity to explore the potential gender differences. Forth, low *Cronbach*’s alpha estimates for AAQ-II, CPQ, and BFI-10 and questionable test–retest reliability for some parts of the Persian version of the SCID-5 may raise doubt about the reliability of these measures. Therefore, the finding related to these constructs must be interpreted cautiously. Fifth, in comparison to previous studies, our yielded correlations among the transdiagnostic factors and insomnia severity were relatively high (Table 1). Given their high standard deviations and the existence of a wide range of insomnia symptoms in the clinical sample, the utility of a correlation matrix to draw conclusions might be criticized. However, since the application of the correlation coefficient test on insomniac patients has not been widely accepted, our conclusions were derived from the results of the regression analysis (Table 2), rather than the correlation matrix. Finally, the cross-sectional design used in this study prevents any causal conclusion. Research with longitudinal design is needed to see what factors predict increases and/or the maintenance of symptom severity or what predicts remission of insomnia symptoms. Longitudinal research adds to our results and help designing an empirical framework to develop more accurate scientific and practical interventions for treating sleep disorders.

## 5. Conclusion

Our findings underlined the importance of transdiagnostic factors of (in descending order) physical concerns (ASI), repetitive negative thinking (RTQ), neuroticism (BFI), personal standards (CPQ), cognitive reappraisal (ERQ), cognitive concerns (ASI), and evaluative concerns (CPQ) in predicting insomnia severity among patients with chronic insomnia. The transdiagnostic factors of physical concerns (ASI) and repetitive negative thinking (RTQ) accounted for the most significant degree of variance in predicting insomnia severity after controlling for demographic characteristics, depression, and anxiety symptoms. Our findings can be applied for the development of intervention programs targeting transdiagnostic factors that have a greater weight in the augmentation of insomnia severity.

## Data availability statement

The original contributions presented in the study are included in the article/supplementary material, further inquiries can be directed to the corresponding authors.

## Ethics statement

The studies involving human participants were reviewed and approved by the ethics committee at the Iran University of Medical Sciences (approval code = IR.IUMS.REC.1397.1346). The patients/participants provided their written informed consent to participate in this study.

## Author contributions

MA and HD: conceptualization, design, and methodology. PS and FH: data collection. MA: formal analysis, funding acquisition, and supervision. MA, HD, and FH: investigation and project administration. HD, PS, and RA: writing the original draft. HD, PS, CB, SM, and MA: revising the manuscript. All authors have contributed to the conception and design of the study, drafted or have been involved in revising this manuscript, reviewed the final version of this manuscript before submission, and agreed to be accountable for all aspects of the work.

## Funding

This research was supported by grant number 97–4–23-13-762 from School of Behavioral Sciences and Mental Health, Iran University of Medical Sciences, received by MA. MA’s time on this research was funded by the Department of Psychology, Norwegian University of Science and Technology, Trondheim, Norway.

## Conflict of interest

The authors declare that the research was conducted in the absence of any commercial or financial relationships that could be construed as a potential conflict of interest.

## Publisher’s note

All claims expressed in this article are solely those of the authors and do not necessarily represent those of their affiliated organizations, or those of the publisher, the editors and the reviewers. Any product that may be evaluated in this article, or claim that may be made by its manufacturer, is not guaranteed or endorsed by the publisher.
